# Dynamic Functional Remodeling of Microglia and Monocyte-Derived Macrophages After Ischemic Stroke: A Narrative Review of Traditional Chinese Medicine Mechanisms and Translational Perspectives

**DOI:** 10.3390/biology15211919

**Published:** 2026-10-23

**Authors:** Xinyu Li, Cuicui Chang, Yujie Kuai, Yanju Gong, Hongfei Li, Xiang Li, Yushi Li, Hangcheng Jiang, Liangqin Shi

**Affiliations:** 1 Chengdu University of Traditional Chinese Medicine, Chengdu 611137, China; 2 School of Medical and Life Sciences, Chengdu University of Traditional Chinese Medicine, Chengdu 611137, China; 3 School of Clinical Medicine, Chengdu University of Traditional Chinese Medicine, Chengdu 611137, China; 4 School of Basic Medical Sciences, Chengdu University of Traditional Chinese Medicine, Chengdu 611137, China; 5 College of Medical Technology, Chengdu University of Traditional Chinese Medicine, Chengdu 611137, China

**Keywords:** ischemic stroke, microglia, monocyte-derived macrophages, myeloid functional states, neuroinflammation, traditional Chinese medicine

## Abstract

Ischemic stroke elicits a dynamic and heterogeneous myeloid response involving brain-resident microglia and recruited monocyte-derived macrophages, whose functional programs are shaped by lineage, spatial context, metabolic state, and disease stage. Traditional Chinese medicine (TCM) has been widely investigated for ischemic stroke, and accumulating evidence suggests that TCM-associated bioactive compounds and multi-component formulas modulate neuroinflammation, metabolism, phagocytosis, and tissue repair through these myeloid populations. However, many studies still interpret treatment effects within the M1/M2 polarization framework, which does not adequately capture post-stroke myeloid complexity. This review reframes TCM-mediated myeloid regulation in terms of dynamic functional remodeling. We examine the distinct origins, spatial heterogeneity, and stage-dependent organization of microglia and monocyte-derived macrophages, then evaluate pharmacological regulation across receptor-mediated sensing, intracellular inflammatory and metabolic signaling, and nuclear transcriptional and epigenetic control. Formula-level evidence is further considered in relation to blood–brain barrier preservation, inflammatory resolution, phagocytic clearance, neural repair, remyelination, and neurological recovery. Current evidence supports multilevel regulation but remains limited by insufficient lineage resolution, reliance on polarization-associated markers, uncertain target-cell accessibility and target engagement, and incomplete demonstration of causal myeloid mediation. Future studies integrating longitudinal, lineage-resolved, and spatial approaches with direct assessment of cellular exposure and target engagement are needed to define when, where, and through which myeloid populations TCM contributes to post-stroke repair.

## Simple Summary

Ischemic stroke occurs when blood flow to part of the brain is blocked, causing brain injury and potentially long-term disability. Immune cells within the brain and those recruited from the bloodstream play important roles in both injury and recovery. Their functions vary according to the stage of stroke and the surrounding tissue environment. Traditional Chinese medicine contains natural compounds and herbal formulas that may influence these immune responses through multiple biological processes. This review examines how these interventions may regulate inflammation, cellular metabolism, the clearance of damaged tissue, and brain repair. Rather than categorizing immune cells as simply harmful or beneficial, we emphasize their diverse and changing functions after stroke. Although experimental studies suggest that certain traditional Chinese medicine interventions may influence these processes, most mechanistic evidence comes from laboratory and animal models. Further research is needed to identify active compounds, clarify their effects on specific immune cell populations, and determine whether these mechanisms contribute to meaningful clinical benefits.

## 1. Introduction

Ischemic stroke remains a major cause of death and long-term neurological disability worldwide [1]. Intravenous thrombolysis and endovascular thrombectomy have substantially improved reperfusion and functional outcomes in eligible patients, but their clinical application remains constrained by treatment windows, imaging characteristics, and patient selection criteria [2]. Moreover, restoration of cerebral blood flow does not immediately terminate tissue injury. Ischemia and reperfusion initiate secondary pathological processes involving oxidative stress, blood–brain barrier disruption, neuroinflammation, and progressive neuronal damage [3]. Consequently, limiting secondary injury while promoting endogenous repair and long-term neurological recovery remains a major therapeutic challenge after ischemic stroke.

Neuroinflammation is a major component of both tissue injury and repair after ischemic stroke, and its biological consequences evolve substantially across the disease course [4]. Brain-resident microglia and infiltrating monocyte-derived macrophages are central components of this response. Resident microglia rapidly sense ischemia-induced alterations in the local microenvironment, whereas blood–brain barrier disruption and cerebrovascular activation promote the recruitment of circulating monocytes that subsequently differentiate into macrophages within the injured brain [5,6]. These populations participate not only in inflammatory amplification but also in debris clearance, metabolic adaptation, vascular stabilization, trophic support, and tissue remodeling. Recent single-cell and spatial transcriptomic studies have further revealed substantial heterogeneity within resident and recruited myeloid populations, with functional programs varying according to cellular origin, anatomical location, metabolic configuration, and disease stage [6]. Post-stroke myeloid responses should therefore be understood as a dynamic and multidimensional functional landscape rather than as a uniform transition between predefined inflammatory and reparative phenotypes.

Traditional Chinese medicine has been extensively investigated as a pharmacological approach for limiting ischemic injury and promoting neurological recovery. Experimental evidence indicates that bioactive constituents and classical formulas can influence inflammatory signaling, oxidative stress, cellular metabolism, cell death, and tissue repair while simultaneously altering phenotypic and functional features of microglia and monocyte-derived macrophages [7,8]. These effects involve interconnected regulatory processes extending from membrane receptor-mediated sensing to intracellular inflammatory and metabolic signaling and nuclear transcriptional control [9,10]. However, much of the existing literature continues to interpret treatment-associated myeloid changes using a restricted set of M1- and M2-associated markers, frequently at a single time point or in whole-brain tissue. Such approaches provide limited information about which myeloid population is affected, whether the observed phenotype corresponds to a defined cellular function, and how treatment effects vary across the evolving post-stroke microenvironment. A mechanistic interpretation of traditional Chinese medicine-mediated myeloid regulation therefore requires moving beyond marker-based polarization toward a framework that incorporates lineage identity, spatial context, temporal dynamics, metabolism, and functional outcome.

From this perspective, pharmacological modulation should be evaluated not simply by changes in a limited set of inflammatory or repair-associated markers, but by whether defined interventions alter biologically relevant myeloid functions and contribute to tissue recovery. In this review, we integrate recent advances in post-stroke myeloid biology with evidence on TCM-associated bioactive compounds and classical formulas. We examine receptor-mediated sensing, intracellular inflammatory and metabolic signaling, nuclear transcriptional regulation, and their relationships with phagocytosis, blood–brain barrier preservation, neural plasticity, white matter repair, and neurological recovery. Particular attention is given to therapeutic timing, lineage specificity, target-cell accessibility, molecular target engagement, and the causal contribution of myeloid remodeling to treatment benefit. By integrating these dimensions, we aim to establish a mechanistically grounded framework for understanding pharmacological regulation of post-stroke myeloid functional states and to identify the experimental evidence required for future translational validation.

### Literature Search Strategy

A literature search was conducted using PubMed to identify studies relevant to the dynamic functional remodeling of microglia and monocyte-derived macrophages after ischemic stroke and their potential regulation by traditional Chinese medicine. The search included combinations of terms related to ischemic stroke, cerebral ischemia–reperfusion injury, microglia, monocyte-derived macrophages, macrophage polarization, traditional Chinese medicine, Chinese herbal medicine, herbal formulas, and bioactive compounds. Additional search terms addressing neuroinflammation, immunometabolism, phagocytosis, blood–brain barrier integrity, and tissue repair were used to capture studies relevant to the molecular mechanisms and functional outcomes discussed in this review. The literature search primarily covered studies published between January 2016 and October 2026, with particular emphasis on research published during the preceding five years. Earlier seminal studies were also considered when they provided essential conceptual or mechanistic foundations for understanding post-stroke myeloid responses.

Original research articles were prioritized, particularly studies investigating post-stroke myeloid heterogeneity, cellular function, and the pharmacological effects of traditional Chinese medicine-associated compounds or formulas. Studies using cellular models, experimental animals, and clinical populations were considered to capture different levels of evidence. Particular attention was given to investigations incorporating pathway inhibition, genetic perturbation, lineage tracing, or functional assays that could strengthen mechanistic interpretation. Publications with limited relevance to ischemic stroke or insufficient evidence to support the discussed biological mechanisms were not prioritized. Additional relevant publications were identified through examination of reference lists.

This review was conducted as a narrative synthesis rather than a systematic review or meta-analysis. The selected evidence was critically interpreted according to experimental context, cellular specificity, mechanistic validation, and translational relevance. Findings derived from cellular and animal models were distinguished from clinical observations, and changes in polarization-associated markers were not considered sufficient evidence of causal myeloid functional remodeling.

## 2. Dynamic Functional Remodeling of Post-Stroke Myeloid Cells

### 2.1. Lineage Identity of Resident Microglia and Monocyte-Derived Macrophages

Following ischemic stroke, the cerebral myeloid response is jointly shaped by brain-resident microglia and peripherally recruited monocyte-derived macrophages. These two populations are ontogenetically distinct. Microglia originate from embryonic yolk sac erythromyeloid progenitors and are maintained throughout adulthood predominantly through local self-renewal, whereas circulating monocytes arise from the adult hematopoietic system [11]. As resident immune sentinels of the brain parenchyma, microglia rapidly respond to ischemia-induced alterations in the local microenvironment [12]. In parallel, disruption of the blood–brain barrier and activation of the cerebrovascular endothelium facilitate the recruitment of peripheral myeloid cells into the injured brain. By day 3 after stroke, venous endothelial populations with high ICAM-1 expression increase in abundance and are associated with enhanced myeloid cell infiltration [13]. The CCL2/CCR2 axis represents a major chemotactic pathway governing the recruitment of circulating monocytes to ischemic tissue [14]. Thus, although their functional programs may partially converge within the injured brain, these two populations remain distinguishable by developmental origin, tissue residency, and route of recruitment.

After entering the ischemic brain, recruited monocytes differentiate into macrophages and, together with activated microglia, undergo extensive functional remodeling in response to the evolving local milieu. In vivo lineage-tracing studies have shown that CCR2-high monocytes recruited during the early phase of stroke can undergo substantial functional reprogramming in response to local cytokine cues, including IL-4 and IL-13 [15]. Importantly, lineage identity does not impose a fixed functional outcome. Microglia contribute to the clearance of necrotic cells and cellular debris, yet under specific conditions they may also engulf injured but viable neurons and thereby aggravate neuronal loss [16]. Similarly, monocyte-derived macrophages cannot be regarded as a uniformly pro-inflammatory population, as IL-33/ST2-dependent macrophage responses have been shown to preserve blood–brain barrier integrity and limit ischemic injury during the early phase of stroke [17]. Collectively, these observations indicate that cellular origin defines lineage identity, whereas functional behavior emerges from the interaction between lineage, disease stage, and the local microenvironment. Distinguishing resident microglia from monocyte-derived macrophages is therefore essential for interpreting post-stroke myeloid heterogeneity and for avoiding the attribution of shared phenotypic markers to biologically equivalent cell states.

### 2.2. Beyond M1/M2 Polarization Toward a Multidimensional Functional-State Continuum

The M1/M2 polarization framework has long provided a convenient conceptual model for describing the opposing functional tendencies of microglia and macrophages. In classical in vitro systems, IFN-γ and lipopolysaccharide predominantly induce pro-inflammatory M1-like responses, whereas IL-4 and IL-13 promote M2-like programs associated with inflammation resolution and tissue repair [18]. However, these experimentally induced states do not adequately represent the complexity of myeloid responses within the post-stroke brain. In vivo, microglia and macrophages rarely conform to mutually exclusive polarization programs. Individual cells may simultaneously display transcriptional and functional features traditionally assigned to different polarization states, and the expression of a limited set of canonical markers does not necessarily predict biological function [19]. The terms M1-like and M2-like are therefore more appropriately used as descriptive indicators of functional tendencies under defined experimental conditions rather than as evidence of discrete and stable cellular identities.

The limitations of the binary framework become particularly evident when the spatial organization of the post-ischemic myeloid response is considered. Integrated single-cell RNA sequencing and spatial transcriptomic analyses have revealed marked differences between microglia located in the ischemic core and those occupying the peri-infarct penumbra. Core-associated microglia exhibit enhanced glycolytic activity together with stronger cytokine and chemokine responses, whereas penumbra-associated microglia show greater reliance on oxidative metabolism, relatively attenuated inflammatory activity, and transcriptional programs associated with myelin support [20]. Functional heterogeneity is also evident among transcriptionally defined microglial populations. CH25H-positive microglia display enhanced phagocytic activity and neuroprotective properties, whereas OASL-positive microglia are enriched in interferon-responsive programs and are associated with greater post-stroke inflammation and infarct severity [21]. This spatial and transcriptional heterogeneity indicates that post-stroke microglial responses cannot be adequately represented along a single inflammatory to reparative axis. Instead, they involve multiple interacting dimensions that include metabolism, inflammatory signaling, phagocytosis, and tissue-support functions.

Monocyte-derived macrophages show a similarly complex pattern of functional diversification. Lineage origin and functional state are closely related but should not be regarded as equivalent. Stroke-associated myeloid cells with prominent lipid-sensing and phagocytic programs can arise from both resident microglia and infiltrating monocytes or monocyte-derived macrophages, indicating that cells of different developmental origins may partially converge on similar functional programs within the ischemic microenvironment [22]. Conversely, monocyte-derived macrophages can acquire states that are not readily accommodated within the conventional M1/M2 framework. FOXP3-positive macrophages, for example, exhibit enhanced clearance of apoptotic cells and a greater capacity to promote the resolution of inflammation [23]. Post-stroke myeloid heterogeneity is therefore better understood as a multidimensional and dynamically evolving functional landscape in which cellular origin, spatial niche, metabolic configuration, and local signaling cues collectively shape cellular behavior. Such a framework provides a more biologically faithful basis for interpreting myeloid responses and their therapeutic modulation than assigning cells to fixed M1-like or M2-like categories.

### 2.3. Stage-Dependent Reorganization of Myeloid Functional States After Ischemic Stroke

The functional landscape of post-stroke myeloid cells evolves continuously as tissue injury, inflammation, and repair proceed. Rather than undergoing a uniform transition from an inflammatory to a reparative phenotype, microglia and monocyte-derived macrophages redistribute among multiple functional states whose relative abundance and biological significance vary over time. During the acute phase, resident microglia rapidly depart from homeostasis and activate inflammatory and immune recruitment programs within hours of ischemic injury. By day 3, proliferative activity becomes increasingly prominent [24]. Single-cell transcriptomic analyses further demonstrate substantial heterogeneity during this early period. Distinct microglial populations coexist with varying degrees of inflammatory activation, including populations enriched in M1-associated inflammatory features and other states that do not conform to canonical M1/M2 transcriptional programs [25]. Peripheral monocytes are recruited concurrently and progressively reshape the local myeloid compartment [22]. The acute post-stroke response should therefore be viewed as an inflammation-dominant but functionally heterogeneous state landscape rather than as a uniform conversion toward a single pro-inflammatory phenotype.

As stroke progresses into the subacute and reparative phases, the balance among myeloid functional programs changes substantially. Recruitment of peripheral monocytes reaches its highest level at approximately day 3. By day 7, pro-inflammatory and repair-associated features coexist among monocyte-derived macrophages, with repair-associated programs becoming increasingly prominent over the following two weeks [26]. Importantly, these temporal changes have functional consequences. Early inhibition of CCR2-dependent monocyte recruitment reduces the expression of anti-inflammatory genes and impairs long-term neurological recovery, indicating that recruited macrophages cannot be regarded as uniformly detrimental even during the early stages of stroke [26]. Resident microglia undergo a parallel functional reorganization. Between days 7 and 30, CD11c-positive microglia progressively accumulate and display enhanced phagocytosis of myelin debris together with an increased capacity to support myelin repair. Selective depletion of this population compromises white matter restoration and functional recovery [27]. The concurrent remodeling of macrophage and microglial programs suggests that recovery is supported by a progressive redistribution of inflammatory, phagocytic, metabolic, and tissue-support functions according to changing tissue demands.

The functional significance of individual markers also varies with disease stage and cellular origin. Arg1 is widely used as an indicator of anti-inflammatory or reparative myeloid responses. However, selective deletion of Arg1 in infiltrating macrophages has been shown to improve functional recovery while reducing fibrosis and synaptic loss after stroke [28]. This apparent paradox illustrates why marker expression alone cannot be assumed to reflect beneficial function. During the chronic phase, differences between resident and recruited myeloid populations become even more pronounced. Lineage-tracing and single-cell transcriptomic analyses have shown that infiltrating monocyte-derived macrophages remain transcriptionally distinct from microglia at days 7 and 14 but are no longer persistently detectable by day 30 in the examined model [29]. These observations indicate that the relative representation of resident and recruited myeloid populations is not fixed across the post-stroke course. Monocyte-derived macrophages can become prominent within the acute ischemic core, whereas their relative representation declines during later recovery as resident microglia persist within the injured brain [29]. In contrast, resident microglia can remain chronically displaced from their homeostatic state. Persistent activation is accompanied by cholesterol accumulation and metabolic reprogramming, resulting in a foam cell-like phenotype associated with impaired white matter recovery. Reducing the cholesterol burden of these microglia promotes tissue repair and functional improvement [30]. Experimental and clinical observations further indicate that altered microglial activity may persist for weeks to months after stroke [30].

**Figure 1:**
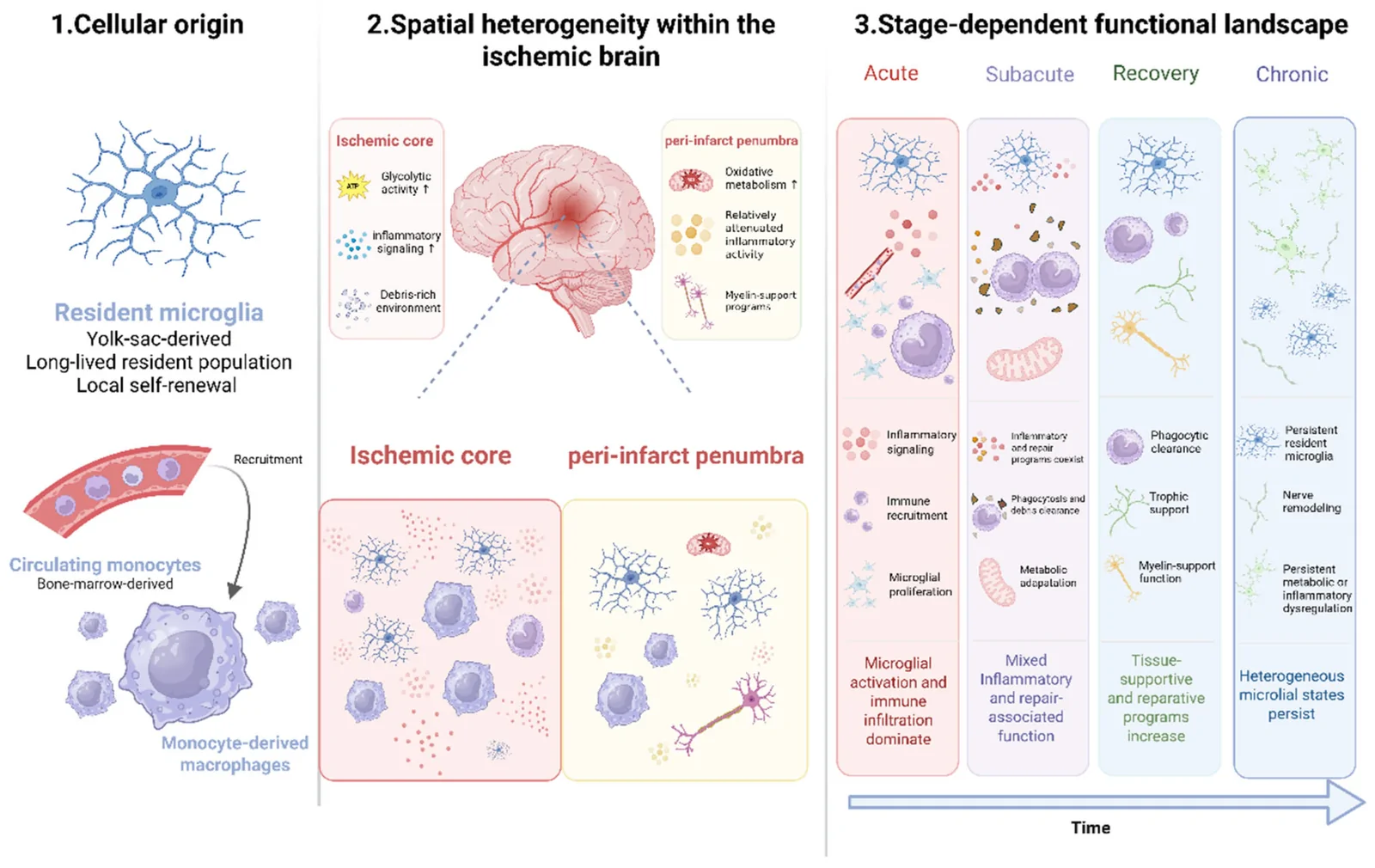
**Temporal dynamics and functional heterogeneity of resident microglia and monocyte-derived macrophages after ischemic stroke.** Resident microglia and infiltrating monocyte-derived macrophages undergo dynamic and partially overlapping changes in functional state across the acute, subacute, recovery, and chronic phases after stroke. Inflammatory activation, proliferation, phagocytosis, metabolic adaptation, trophic support, and repair-associated programs vary according to disease stage, spatial context, local microenvironment, and cellular origin. These responses constitute a multidimensional functional-state landscape rather than a simple transition between predefined inflammatory and reparative polarization states. Created in BioRender. Xinyu Li. (2026) [https://BioRender.com] (https://BioRender.com).

Taken together, post-stroke myeloid responses are characterized by stage-dependent reorganization rather than a predetermined transition from inflammatory to reparative polarization states. Cellular origin, spatial context, metabolic state, and evolving tissue requirements jointly determine whether a given myeloid program contributes to injury containment, inflammatory resolution, debris clearance, or tissue repair. Consequently, the biological significance of myeloid modulation can only be interpreted within a defined temporal context. This temporal dependence also has important therapeutic implications because an intervention that is beneficial during one stage of stroke may not exert the same effect when applied at another stage (Figure 1).

## 3. Molecular Mechanisms of TCM-Associated Bioactive Compounds in Post-Stroke Myeloid Remodeling

Post-stroke myeloid states emerge from the continuous integration of extracellular injury signals with intracellular signaling, metabolic programs, and transcriptional control. Ischemia-derived cues are detected at the cell surface, propagated through cytoplasmic signaling networks and metabolic sensors, and ultimately translated into nuclear programs that shape inflammatory activity, phagocytosis, metabolic adaptation, and tissue repair. Current evidence suggests that TCM-associated bioactive compounds can intervene at multiple points along this regulatory hierarchy. However, the strength of mechanistic evidence varies considerably among individual targets, and changes in polarization-associated markers alone do not establish causal reprogramming of myeloid cell states. Accordingly, this section examines TCM-mediated myeloid remodeling as an integrated process involving receptor-mediated sensing, intracellular inflammatory and metabolic integration, and nuclear transcriptional and epigenetic control. This framework focuses on how pharmacological perturbations alter myeloid functional programs rather than simply reclassifying treatment effects according to M1- or M2-associated markers.

### 3.1. Receptor-Mediated Sensing of Ischemic Signals

Membrane receptors form the first regulatory interface through which microglia and macrophages interpret the ischemic microenvironment. Damage-associated molecular patterns and extracellular ATP activate receptors including TLR4 and P2X7R, which can sustain inflammatory signaling when excessively or persistently engaged. Astragalus polysaccharides suppress P2X7R-associated signaling, accompanied by reduced pro-inflammatory features and increased repair-associated markers [31]. The functional relevance of P2X7R is further supported by receptor activation experiments showing that increased P2X7R activity suppresses repair-associated microglial responses and aggravates neuroinflammation after stroke [32]. The convergent pharmacological and receptor-activation evidence suggests that limiting persistent damage-sensing signals can alter the upstream conditions that favor the establishment and maintenance of inflammatory myeloid programs.

TREM2 represents a functionally distinct receptor axis that is more closely associated with phagocytosis, metabolic adaptation, and tissue repair. Bone marrow chimera experiments indicate that preservation of TREM2 in resident microglia enhances phagocytic activity and is associated with reduced ischemic injury and improved neurological recovery, whereas TREM2 expressed by peripheral myeloid cells appears to make a more limited contribution [33]. Single-nucleus transcriptomic analyses further show that microglia with high TREM2 expression exhibit enhanced phagocytic and oxidative metabolic programs, while Trem2 deficiency compromises these functions and exacerbates ischemic injury [34]. Baicalin provides additional pharmacological evidence for the involvement of this pathway. Baicalin increases TREM2 expression and suppresses pro-inflammatory phenotypes, while TREM2 knockdown substantially weakens these effects and TREM2 overexpression further enhances them [35]. TREM2 knockdown does not completely abolish changes in repair-associated phenotypes. This finding suggests that TREM2 functions as an important regulatory node within a broader functional network rather than as a determinant of a discrete reparative myeloid state.

Collectively, membrane receptor modulation appears to reshape the upstream conditions that govern post-stroke myeloid responses rather than directly convert cells between predefined polarization states. Suppression of excessive TLR4 and P2X7R signaling may constrain persistent inflammatory input, whereas TREM2 is more closely linked to phagocytic, metabolic, and repair-associated functions. However, direct receptor binding, cell-specific target engagement, and genetic validation remain limited for most TCM-associated bioactive compounds. Receptor-associated changes should therefore be interpreted cautiously because they may reflect direct pharmacological action, indirect remodeling of the ischemic microenvironment, or both. These upstream signals subsequently converge on intracellular signaling and metabolic networks, where they are further integrated into functional cellular responses.

### 3.2. Intracellular Signaling and Metabolic Integration

Within the cytoplasm, inflammatory signaling intersects with metabolic regulation to shape myeloid functional states. NF-κB represents a central signaling node in this process because sustained NF-κB activation maintains the transcription of multiple inflammatory mediators after ischemic injury. Tanshinone IIA reduces the phosphorylation of IκBα and NF-κB p65, accompanied by decreased expression of CD32 and iNOS and increased expression of Arg1 and CD206 [36]. In vivo immunofluorescence analyses further show a reduction in CD16/32-positive microglia and an increase in CD206-positive microglia, supporting an association between suppression of NF-κB activity and changes in polarization-associated markers. Hydroxysafflor yellow A acts through an upstream regulatory mechanism by enhancing SIRT1 activity, reducing HMGB1 acetylation and cytoplasmic translocation, and suppressing NF-κB p65 activation [37]. Both genetic and pharmacological inhibition of SIRT1 weaken these effects, providing stronger evidence that the SIRT1/HMGB1/NF-κB axis contributes functionally to microglial state regulation. PI3K/Akt signaling also participates in the integration of inflammatory and survival signals. Erianin alters PI3K/Akt-associated signaling while reducing NF-κB p65 phosphorylation and nuclear translocation, accompanied by attenuation of pro-inflammatory phenotypes and increased expression of repair-associated markers [38]. However, direct evidence that PI3K/Akt activity is required for these changes remains less complete. Current findings therefore support NF-κB and its upstream regulators more strongly than PI3K/Akt as mechanistically validated determinants of TCM-associated changes in myeloid phenotypes.

Intracellular regulation extends beyond canonical inflammatory signaling because the functional capacity of myeloid cells is closely coupled to cellular metabolism. AMPK serves as a major energy sensor that coordinates metabolic availability with inflammatory responses. In cerebral ischemia and reperfusion models, aucubin increases CD206 and Arg1 expression while reducing Nos2 expression and inflammatory activity. Pharmacological inhibition of AMPK markedly attenuates these effects, supporting a functional contribution of AMPK to the regulation of polarization-associated phenotypes [39]. Formononetin provides a more direct link between intracellular signaling and metabolic reprogramming. Treatment reduces glucose uptake and lactate production, suppresses glycolysis, and enhances mitochondrial oxidative metabolism in microglia. These metabolic changes occur together with reduced pro-inflammatory polarization and increased repair-associated phenotypes [40]. AMPK inhibition weakens both the metabolic effects and the associated phenotypic changes. These findings suggest that AMPK-mediated myeloid regulation cannot be explained solely by suppression of inflammatory signaling. Instead, restoration of the balance between glycolytic and mitochondrial metabolism may contribute directly to the establishment of distinct functional states. Metabolic reprogramming may therefore represent a mechanistic bridge through which intracellular signaling translates ischemic stress into altered myeloid behavior.

Inflammasome signaling constitutes another component of intracellular integration, although its relationship with myeloid state remodeling requires more cautious interpretation. Activation of the NLRP3 inflammasome promotes caspase-1 activation, maturation of IL-1β and IL-18, and GSDMD-dependent pyroptosis. Curcumin reduces GSDMD, active caspase-1, and mature inflammatory cytokines in post-stroke microglia and macrophages, while NLRP3 knockdown further suppresses pyroptosis, reduces white matter injury, and improves sensorimotor outcomes [41]. Effusol provides stronger evidence for direct molecular target engagement. It suppresses both NLRP3 priming and activation, while DARTS and CETSA analyses identify NLRP3 as a potential molecular target. Additional pharmacological inhibition experiments further support the functional involvement of NLRP3 in the effects of Effusol [42]. Nevertheless, these studies primarily establish regulation of inflammasome activation and pyroptotic injury rather than direct control of myeloid functional state transitions. Classical polarization markers and broader functional programs have been evaluated less extensively in this context. NLRP3 inhibition should therefore be interpreted as modulation of an inflammatory effector pathway that can reshape the cellular environment rather than as definitive evidence of a direct switch between predefined myeloid states.

Overall, intracellular regulation of post-stroke myeloid responses reflects the integration of inflammatory signaling, metabolic adaptation, and cellular stress pathways rather than the activity of a single molecular cascade. Current evidence provides comparatively stronger mechanistic support for NF-κB- and AMPK-associated regulation, whereas the contribution of PI3K/Akt signaling and inflammasome modulation to broader myeloid state remodeling remains less firmly established. Importantly, suppression of inflammation, metabolic reprogramming, and alteration of myeloid functional states are related but not biologically equivalent processes. These cytoplasmic and metabolic signals ultimately converge on nuclear transcriptional programs that shape the persistence and functional consequences of post-stroke myeloid responses.

### 3.3. Nuclear Transcriptional and Epigenetic Regulation

Cytoplasmic signals can influence myeloid states through several nuclear transcriptional routes. Several TCM-associated bioactive compounds influence myeloid responses through signaling pathways that ultimately converge on nuclear transcriptional regulators. Baicalein suppresses pro-inflammatory microglial phenotypes while inhibiting TLR4-dependent NF-κB activation and nuclear translocation, together with reduced STAT1 phosphorylation [43]. Among the repair-associated transcriptional regulators implicated in this process, PPARγ has received relatively strong mechanistic support. Ginkgetin promotes PPARγ nuclear translocation and enhances repair-associated polarization, whereas pharmacological antagonism of PPARγ markedly attenuates these effects [44]. Astragaloside IV similarly increases PPARγ expression and promotes repair-associated phenotypes in microglia and macrophages. PPARγ blockade weakens this phenotypic shift and reduces neurogenesis and angiogenesis [9]. The available antagonist and pathway-intervention data support PPARγ as a transcriptional regulator that links upstream signaling changes to repair-associated myeloid programs rather than as a simple marker of a predefined reparative phenotype.

Nuclear regulation of myeloid states is also closely integrated with cellular redox balance. Fraxin promotes a repair-associated phenotype while enhancing PPARγ and Nrf2-associated signaling and suppressing NF-κB activation. Pharmacological inhibition of PPARγ reduces the Nrf2 response, restores NF-κB activity, and weakens the associated polarization shift, suggesting functional interaction among these transcriptional programs [45]. Nrf2 provides an additional mechanism through which oxidative stress can influence myeloid behavior. Curcumol reduces oxidative stress while enhancing Nrf2-associated signaling and suppressing NF-κB activation in microglia. Both pharmacological inhibition and knockdown of Nrf2 markedly attenuate the polarization-associated effects of curcumol [46]. These observations indicate that redox-responsive transcription actively participates in myeloid state regulation rather than merely reflecting a secondary consequence of reduced oxidative stress. More broadly, the interaction among PPARγ, Nrf2, and NF-κB suggests that TCM-associated bioactive compounds may remodel myeloid function through coordinated regulation of interconnected transcriptional networks rather than through isolated control of a single nuclear factor.

Metabolic reprogramming can also influence myeloid states through changes in the nuclear localization and regulatory activity of metabolic proteins. Under ischemic conditions, PKM2 translocates to the nucleus and interacts with acetylated histone H3 and HIF-1α, thereby promoting pro-inflammatory gene expression [47]. Interference with PKM2 nuclear translocation or its nuclear interactions attenuates pro-inflammatory microglial polarization, supporting a mechanistic connection between metabolic state and transcriptional control. Saponins from *Panax notoginseng* (Burkill) F.H.Chen reduce nuclear accumulation of PKM2 in activated microglia after stroke and concurrently suppress downstream inflammatory signaling [48]. However, the direct requirement of PKM2 nuclear translocation for TCM-induced myeloid state remodeling has not yet been demonstrated within the same experimental system. PKM2 should therefore be regarded as a plausible mechanistic link between metabolic reprogramming and nuclear transcription rather than as a fully validated mediator of TCM-induced state transitions.

Epigenetic and post-translational regulation adds a further layer of control over inflammatory transcription. Pterostilbene inhibits HDAC3, increases Nrf1 acetylation and stability, and suppresses NF-κB-associated inflammatory responses. Experimental manipulation of HDAC3 activity and Nrf1 acetylation supports the functional involvement of this regulatory axis [49]. However, direct evidence linking such nuclear modifications to defined myeloid functional states remains less developed than that available for PPARγ and Nrf2. Collectively, these findings support a hierarchical model in which receptor-mediated sensing and cytoplasmic signaling converge on nuclear transcriptional and epigenetic programs that shape the persistence and functional consequences of post-stroke myeloid responses. Among these mechanisms, PPARγ- and Nrf2-associated regulation currently has stronger mechanistic support, whereas metabolic protein translocation and epigenetic regulation require further causal validation. Representative preclinical evidence across these regulatory levels, together with the corresponding experimental models and evidence types, is summarized in Table 1.

## 4. Multi-Component TCM Formulas in Post-Stroke Myeloid Remodeling and Tissue Repair

Compared with individual bioactive compounds, classical TCM formulas contain multiple pharmacologically active constituents capable of influencing interconnected regulatory processes. Their effects on post-stroke myeloid cells are therefore unlikely to be adequately explained by changes in a single receptor or signaling pathway. Current evidence suggests that different TCM formulas can influence inflammatory signaling, transcriptional regulation, metabolic processes, and functional myeloid responses through partially overlapping mechanisms. Importantly, the biological relevance of such multilevel regulation depends not simply on the number of molecular targets involved, but on whether coordinated changes in these targets are linked to meaningful cellular and tissue outcomes. Because the bioavailable constituents responsible for these effects are often incompletely resolved at the formula level, pathway changes should not automatically be interpreted as evidence of direct constituent-specific target engagement. Accordingly, this section focuses on how TCM formulas influence multiple components of myeloid regulatory networks and how these changes are associated with neural repair, blood–brain barrier preservation, phagocytic clearance, and white matter remodeling.

### 4.1. Coordinated Regulation of Inflammatory and Transcriptional Networks

Classical TCM formulas may remodel post-stroke myeloid states through coordinated regulation of multiple inflammatory and transcriptional nodes. Danhong Injection reduces pro-inflammatory markers and increases repair-associated markers in microglia, accompanied by restoration of JUNB and IκBα expression and suppression of NF-κB activation. JUNB silencing markedly weakens the effects of Danhong Injection on both NF-κB-associated inflammatory responses and polarization-related phenotypes [54]. JUNB silencing therefore provides functional evidence that this transcriptional regulator contributes to formula-associated changes in microglial inflammatory and polarization-related responses rather than merely changing as a secondary consequence of reduced inflammation. In this context, JUNB appears to function as a regulatory node linking upstream intervention to downstream inflammatory and phenotypic responses.

Constituent-level evidence provides a complementary perspective on the mechanisms associated with Danhong Injection. Tanshinone IIA suppresses IκBα and NF-κB p65 phosphorylation [36], whereas hydroxysafflor yellow A regulates SIRT1/HMGB1/NF-κB signaling [37]. These findings suggest convergence on NF-κB-associated inflammatory regulation through distinct upstream mechanisms, consistent with the signaling changes observed following Danhong Injection treatment. However, this mechanistic overlap does not establish the contribution of either constituent to the effects of the complete formula. Comparative studies of individual constituents, their combinations, and the complete preparation are needed to clarify their respective contributions and potential interactions.

Functional evidence for the involvement of specific regulatory nodes has also been reported for Huoluo Xiaoling Pellet. Treatment increases MCPIP1 and PPARγ expression while reducing pro-inflammatory markers and enhancing repair-associated phenotypes. Knockdown of MCPIP1 attenuates the effects of the formula on myeloid polarization, inflammatory responses, and ischemic brain injury [55]. Compared with studies based solely on parallel changes in signaling proteins, this functional perturbation provides stronger support for a mechanistic role of MCPIP1 in the immunomodulatory effects of the formula. Together, the Danhong Injection and Huoluo Xiaoling Pellet studies suggest that formula-mediated myeloid remodeling can involve defined transcriptional regulators that are functionally required for at least part of the observed biological response. The available perturbation data do not yet establish that all molecular changes induced by a formula form a single continuous regulatory cascade.

Upstream cytokine signaling may provide an additional route through which formulas reshape myeloid responses. Naoqing Formula reduces Csf3 expression and JAK2, STAT3, and STAT6-associated signaling, accompanied by changes in macrophage polarization [56]. Parallel findings in cellular models further support the involvement of JAK/STAT signaling in these effects. Nevertheless, the mechanistic evidence is less direct than that available for JUNB and MCPIP1 because the requirement of individual signaling components for the observed myeloid changes has not been established with comparable functional perturbation. The current evidence therefore supports an association between Csf3 and JAK/STAT signaling and formula-induced myeloid remodeling, but does not yet define a fully validated causal pathway.

Overall, current formula-level evidence supports coordinated regulation of multiple inflammatory and transcriptional nodes, but does not yet demonstrate pharmacological synergy. Establishing genuine network-level cooperation will require evidence that distinct components or regulatory nodes make complementary and functionally necessary contributions within relevant myeloid populations. Future studies should determine whether these nodes are causally connected and whether their combined regulation is required for downstream tissue repair. This distinction is essential for moving multi-target regulation from a descriptive feature of TCM formulas toward a mechanistically testable framework.

### 4.2. Myeloid State Remodeling and Neurovascular Unit Repair

During the recovery phase of stroke, the significance of formula-induced myeloid remodeling extends beyond changes in inflammatory markers and becomes increasingly relevant to restoration of the neurovascular unit. Huangqi Guizhi Wuwu Decoction promotes repair-associated microglial phenotypes while increasing dendritic spine density and the expression of synaptic proteins. Inhibition of SIRT1 markedly attenuates the effects of the formula on microglial polarization, synaptic plasticity, and neurological function [57]. The SIRT1 inhibition data support the involvement of SIRT1 in both myeloid responses and neural repair, although they do not establish a myeloid-cell-specific causal link between these processes. Longer-term studies provide further evidence that this relationship persists during recovery. At 21 days after stroke, Huangqi Guizhi Wuwu Decoction continues to enhance repair-associated polarization together with increased expression of GAP43, PSD95, and BDNF and greater axonal and synaptic remodeling. Pharmacological inhibition of PI3K or mTOR partially weakens these effects, whereas Akt activation partially restores them [58]. Across both time points, formula-induced myeloid remodeling was observed in parallel with structural and functional processes that support neural recovery. However, these observations do not yet establish that a specific microglial state is the indispensable cellular mediator of these reparative effects.

The effects of Huangqi Guizhi Wuwu Decoction can be further considered alongside those of astragaloside IV, a bioactive constituent of Astragalus, one of the herbs included in the formula. Astragaloside IV promotes PPARγ-associated myeloid responses and enhances neurogenesis and angiogenesis, with PPARγ antagonism attenuating these effects [9]. In contrast to the SIRT1- and PI3K/Akt/mTOR-associated mechanisms reported for the complete formula [57,58], the constituent-level findings implicate PPARγ, suggesting that distinct regulatory pathways may be associated with overlapping myeloid and tissue repair outcomes. Nevertheless, these separate studies do not establish that astragaloside IV mediates the effects of Huangqi Guizhi Wuwu Decoction or that the identified pathways interact within the formula. Direct comparisons of the constituent and the complete preparation, together with targeted mechanistic validation, are needed to clarify these relationships.

Myeloid remodeling is also associated with restoration of the vascular component of the neurovascular unit. Sanhua Decoction induces a less inflammatory myeloid profile in the ischemic brain, characterized by reduced CD16 and increased CD206 expression. These changes coincide with restoration of the tight junction proteins ZO-1, Occludin, and Claudin-5 and with reduced MMP-9 expression, suggesting an association between myeloid regulation and improved blood–brain barrier integrity [59]. Evidence from an antibiotic-induced pseudo-germ-free model further indicates that the gut microbiota may contribute to this response. Antibiotic treatment attenuates the effects of Sanhua Decoction on selected microglial indicators, including Iba1 and CX3CL1, while also altering microbial composition and short-chain fatty acid metabolism. Nevertheless, depletion of the microbiota does not uniformly abolish the neurological and metabolic benefits of the formula. The available evidence therefore supports an interaction among gut microbial regulation, central myeloid responses, and blood–brain barrier recovery, but does not establish a linear causal pathway in which microbiota-driven microglial polarization directly mediates vascular protection.

These studies place myeloid remodeling at the interface between TCM intervention and neurovascular repair. Formula-induced changes in myeloid states are consistently accompanied by synaptic plasticity, axonal remodeling, blood–brain barrier stabilization, and neurological recovery. Pharmacological perturbation of SIRT1 and PI3K/Akt/mTOR supports the involvement of these signaling pathways in both myeloid and reparative responses, but current evidence does not establish that myeloid remodeling is required for the observed tissue-level benefits. Resolving this relationship will require lineage-resolved manipulation of myeloid populations together with direct assessment of neuronal and vascular function to determine whether defined myeloid states act as causal intermediates between formula exposure and neurovascular recovery.

### 4.3. Stage-Dependent Functional Priorities from Acute Injury Control to White Matter Repair

The functional significance of TCM-mediated myeloid regulation varies substantially across the course of stroke. During the early injury phase, excessive inflammatory amplification and cellular stress represent major therapeutic concerns, whereas during recovery, effective clearance of damaged tissue and provision of trophic support become increasingly important. Myeloid modulation should therefore be evaluated according to the functional demands of each disease stage rather than interpreted solely through changes in pro-inflammatory or repair-associated markers.

During the early phase of injury, Buyang Huanwu Decoction has been shown to regulate inflammatory and cell injury-associated responses in a delayed tissue-type plasminogen activator (t-PA) thrombolysis model. Treatment increases microglial PGC-1α expression while reducing NF-κB nuclear translocation, NLRP3 inflammasome activation, and pyroptosis, together with changes toward a more repair-associated polarization profile [60]. Silencing of PGC-1α markedly attenuates the inhibitory effects of the formula on inflammasome activation and pyroptosis-related proteins, supporting a functional role for PGC-1α in this regulatory response. However, these experiments do not establish myeloid state remodeling as the direct intermediate responsible for reduced pyroptotic injury. The findings are therefore more appropriately interpreted as coordinated modulation of inflammatory myeloid responses and cellular injury rather than as evidence that a polarization shift itself suppresses pyroptosis.

A different functional pattern becomes evident during the recovery phase. At 28 days after stroke, Buyang Huanwu Decoction improves white matter microstructural integrity and myelin preservation while enhancing microglial interaction with myelin debris and increasing phagocytosis-associated signatures including AXL and CD11c [61]. Pharmacological inhibition of TREM2 attenuates phagocytosis-associated microglial responses and reduces the white matter protective effects of Buyang Huanwu Decoction, supporting the involvement of TREM2 signaling in post-stroke recovery without fully establishing microglial phagocytosis as the required causal mediator of these benefits. Increased TREM2 signaling is also accompanied by enhanced microglial IGF1 production together with greater oligodendrocyte proliferation and remyelination [61]. These observations extend the interpretation of myeloid regulation beyond conventional polarization markers by linking a defined microglial response to debris clearance, trophic support, and white matter reconstruction.

These stage-specific findings highlight a shift in the functional priorities of myeloid regulation across the post-stroke course. During the early phase, therapeutic benefit is more closely associated with limiting excessive inflammatory signaling, inflammasome activation, and cellular injury. During recovery, relevant myeloid functions increasingly include phagocytic clearance, trophic support, and remyelination. Importantly, evidence for these early and late effects derives largely from separate experimental paradigms, and it remains unclear whether they represent coordinated remodeling within the same myeloid populations over time. Longitudinal studies that preserve cellular identity while tracking functional state and tissue outcome will therefore be necessary to define the temporal organization of formula-mediated myeloid regulation.

**Figure 2:**
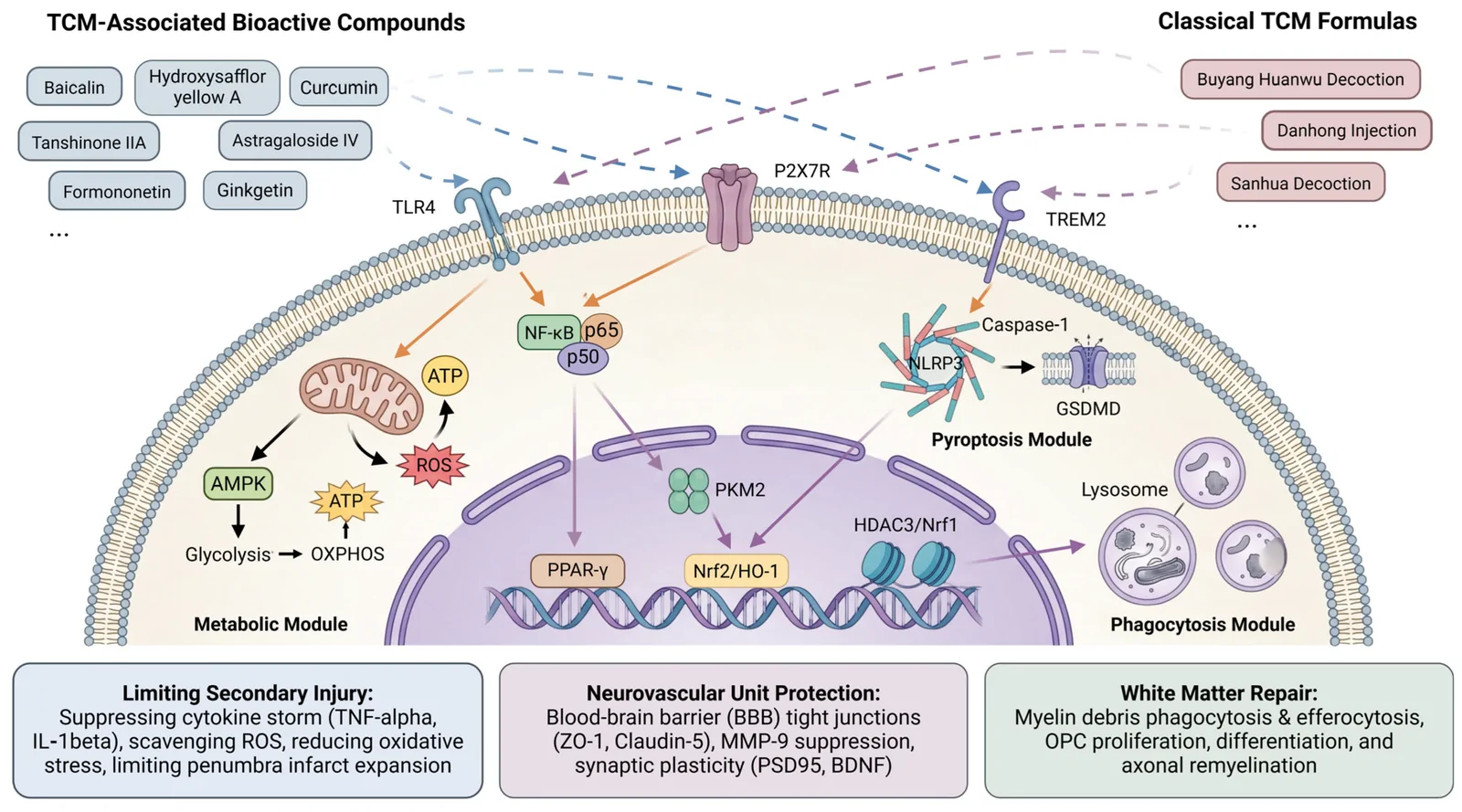
**Multilevel mechanisms underlying TCM-mediated regulation of post-stroke myeloid functional states and associated outcomes.** TCM-associated bioactive compounds and classical TCM formulas influence post-stroke myeloid responses through interconnected regulatory levels involving membrane receptor-mediated sensing, cytoplasmic inflammatory and metabolic signaling, and nuclear transcriptional and epigenetic regulation. These mechanisms reshape inflammatory signaling, metabolic adaptation, phagocytosis, trophic support, and pyroptotic responses, thereby influencing blood–brain barrier integrity, neurovascular remodeling, white matter repair, and neurological recovery. Solid arrows indicate canonical signaling relationships or experimentally supported functional interactions, whereas dashed arrows connecting TCM-associated compounds or formulas to membrane receptors represent reported modulation of receptor-associated signaling rather than experimentally demonstrated direct receptor binding. T-shaped connectors indicate inhibition. Created in BioRender. Xinyu Li. (2026) [https://BioRender.com] (https://BioRender.com).

Current studies of multi-component TCM formulas therefore support a more function-oriented interpretation of post-stroke myeloid regulation that extends beyond conventional polarization markers. Formula-associated changes in myeloid responses have been linked to neural plasticity, blood–brain barrier preservation, phagocytic clearance, trophic support, white matter repair, and neurological recovery, but the causal hierarchy connecting formula exposure, myeloid remodeling, and tissue recovery remains incompletely resolved. Establishing this relationship will require cell-specific and longitudinal approaches capable of distinguishing mechanistically relevant myeloid responses from parallel features of broader neuroprotection. An integrated overview of these molecular and functional relationships is presented in Figure 2 and representative formula-level studies are summarized in Table 2.

## 5. Critical Evidence Gaps and Translational Priorities

Evidence for TCM-mediated regulation of post-stroke myeloid responses has progressed from descriptive changes in polarization-associated markers toward increasingly detailed analyses of signaling, metabolism, and cellular function. However, three major gaps continue to limit mechanistic interpretation: insufficient temporal resolution, inadequate distinction between resident and recruited myeloid populations, and uncertain target-cell engagement. These limitations are particularly important because similar phenotypic features may have different functional consequences depending on disease stage, cellular origin, and local tissue context. Resolving these gaps is therefore essential for determining whether TCM interventions produce stage-appropriate and cell-specific myeloid remodeling that contributes causally to tissue repair and functional recovery.

### 5.1. From Single-Time-Point Snapshots to Stage-Matched Intervention

The therapeutic significance of myeloid modulation depends not only on the direction of a phenotypic change but also on when that change occurs. Post-stroke myeloid responses do not progress through a predetermined sequence from inflammation to repair. Instead, cellular composition, transcriptional programs, metabolic activity, and functional properties are continuously reorganized as the requirements of the injured brain evolve. Longitudinal single-cell analyses demonstrate substantial changes in microglial populations between days 3, 7, and 14 after stroke, while SPP1-associated activation and antigen-presentation programs can remain detectable during the later stages of this period [64]. These observations indicate that the persistence of an activated cell population does not necessarily imply persistence of the same functional state.

Temporal remodeling also involves changes in the composition and interaction of resident and recruited myeloid populations. Transient alterations in microglial niches during the early phase can facilitate the recruitment of CCR2-positive monocyte-derived macrophages with elevated GPNMB and CD63 expression, enhanced lipid metabolism, and pro-angiogenic properties. Transplantation experiments support a reparative role for these cells, while MafB contributes to maintenance of their transcriptional program [65]. Similarly, transient microglial depletion followed by endogenous repopulation alters both the properties of newly generated microglia and the recruitment trajectories of peripheral myeloid cells [66]. These temporal perturbation studies illustrate that an intervention applied at a particular stage may modify not only the state of existing cells but also the composition and subsequent evolution of the myeloid compartment.

The relationship between cellular persistence and functional benefit becomes even more complex during the chronic phase. Reparative microglial populations may remain within the injured brain while progressively losing their recovery-associated transcriptional properties. Increased ZFP384 expression disrupts YY1-dependent chromatin interactions and contributes to erosion of these programs, whereas targeting Zfp384 during the chronic stage preserves reparative potential and improves functional recovery [67]. Thus, the continued presence of a nominally reparative population does not guarantee continued reparative function. Therapeutic evaluation based on a single marker or a single time point may therefore fail to distinguish transient phenotypic changes from sustained and functionally meaningful remodeling.

These findings argue against assigning a uniform therapeutic value to identical marker changes across the post-stroke course. Reductions in CD86 or increases in CD206 and Arg1 may have different biological implications depending on cellular composition, tissue context, and functional demands at the time of intervention. Therapeutic windows should therefore be defined by the alignment of treatment exposure with myeloid composition and functional demands rather than by chronological time alone. Longitudinal studies should distinguish transient marker shifts from sustained functional remodeling and determine whether therapeutic benefit is restricted to specific biological windows.

### 5.2. Lineage Resolution and Interorgan Regulation of Myeloid Responses

Therapeutic timing alone cannot resolve which myeloid populations mediate the effects of TCM interventions. Resident microglia and monocyte-derived macrophages can acquire overlapping inflammatory, phagocytic, and repair-associated programs after stroke, making treatment-associated changes difficult to assign to a specific lineage. This limitation is particularly relevant when conventional polarization markers are measured in whole-brain tissue or incompletely resolved myeloid populations, because similar phenotypic changes may arise from resident microglia, recruited macrophages, or both. Establishing cellular origin is therefore essential for determining where and through which myeloid populations TCM-mediated regulation occurs.

Single-cell technologies have substantially improved the cellular resolution of mechanistic studies, but improved localization should not be equated with demonstration of cell-specific causality. In studies of Shuanglu Tongnao Formula, single-cell RNA sequencing revealed altered communication between microglia and other immune populations after ischemic injury. Integration with network pharmacology further associated AGE–RAGE-related signaling with formula-induced changes in myeloid polarization, while alterations in CCND1, IL-1β, and phosphorylated STAT3 were subsequently validated in brain tissue [68]. This integrated analysis identifies candidate pathways and cellular interactions, but does not establish that the formula directly regulates AGE–RAGE signaling within microglia. The observed microglial changes could also arise indirectly through effects on infiltrating immune cells or other components of the ischemic microenvironment. Similar limitations persist even when cellular profiling is combined with target-oriented pharmacology. For Huanglian Jiedu Decoction, single-cell RNA sequencing identified macrophages, microglia, and neutrophils as major pyroptosis-associated populations, while multi-omics analysis, molecular docking, SPR-based affinity assessment, and molecular validation converged on NLRP3 as a candidate therapeutic target [69]. Nevertheless, these multilevel data do not establish whether NLRP3 regulation within a defined myeloid population is required for therapeutic benefit. This distinction illustrates the gap between cell-resolved target association and cell-specific causal validation.

Establishing cell-specific causality requires direct testing of cellular necessity. Lineage-tracing approaches can distinguish resident microglia from recruited monocyte-derived macrophages and define their temporal contributions after stroke [29]. Conditional genetic manipulation and cell-specific pharmacological approaches can then determine whether a candidate pathway is functionally required within a particular lineage. Spatially resolved analyses are also necessary because post-stroke microglia exhibit marked heterogeneity between the ischemic core and peri-infarct regions [20]. Integrating lineage, functional perturbation, and spatial mapping would clarify not only which myeloid populations respond to treatment, but also where these responses occur and whether they are required for downstream neurological and tissue-reparative effects.

Cellular attribution becomes more complex when TCM interventions modify peripheral systems that subsequently influence central myeloid responses. Notoginsenoside R1 ameliorates stroke-associated alterations in gut microbial composition, intestinal barrier integrity, and systemic inflammation. Fecal microbiota transplantation from treated animals partially reproduces its neuroprotective effects and is accompanied by reduced microglial activation and attenuation of TLR4/MyD88/NF-κB signaling in the brain [70]. These findings support a contribution of the gut microbiota to central immune regulation, but they do not identify the microbial products or systemic mediators responsible for this effect, nor do they establish whether a defined microglial functional state is required for neuroprotection.

Further evidence for microbiota-dependent mechanisms comes from TCM formulas that influence defined microbial metabolites. In a rat model of chronic cerebral ischemia, Dengzhan Shengmai increased microbiota-derived short-chain fatty acids, improved intestinal barrier integrity, and attenuated neuroinflammation. Antibiotic treatment weakened several protective effects, whereas fecal microbiota transplantation reproduced beneficial changes, supporting a functional contribution of the gut microbiota [71]. In a middle cerebral artery occlusion model, Buqi-Huoxue-Tongnao Decoction improved intestinal barrier integrity and altered microbial tryptophan metabolism, accompanied by increased indole lactic acid production. Transplantation of treatment-associated microbiota and oral supplementation with indole lactic acid independently improved ischemic outcomes, further implicating microbial metabolites in formula-associated neuroprotection [72]. Together, these studies suggest that TCM interventions may restore aspects of gut–brain axis homeostasis through microbiota-dependent metabolic and barrier-related mechanisms, although their effects on defined post-stroke myeloid functional states remain unresolved.

Despite these advances, mechanistic studies outside the TCM field provide a useful benchmark for resolving specific communication pathways between peripheral signals and cerebral myeloid responses. Gut-derived kynurenic acid, for example, can signal through GPR35-expressing intestinal neurons and vagal pathways to influence post-stroke microglial responses [73]. Although this mechanism has not been demonstrated for TCM interventions, it illustrates how a peripheral mediator can be linked to a defined sensor, communication route, and central myeloid response. TCM studies of the gut–brain axis should similarly distinguish associative changes in microbiota, metabolites, and microglial markers from mechanistically resolved pathways in which the mediator, sensor, communication route, and responding cell population are experimentally defined.

Resolving TCM-mediated myeloid regulation therefore requires distinguishing cellular lineage from the route of regulation. The key questions are whether therapeutic effects are mediated by resident microglia, monocyte-derived macrophages, or both, and whether these populations are regulated directly within the ischemic brain or indirectly through peripheral immune, metabolic, or gut-derived signals. Addressing these questions will require lineage-resolved and spatially resolved analyses combined with functional perturbation of candidate pathways. Such approaches will define the cellular source, anatomical context, and mechanistic route through which TCM exposure influences post-stroke myeloid responses and recovery.

### 5.3. From Tissue-Level Changes to Target-Cell Engagement and Clinical Outcomes

Identification of a candidate molecular target does not establish that a therapeutic agent reaches or engages that target within the relevant myeloid population in vivo. This distinction is particularly important for TCM-associated bioactive compounds, which may undergo extensive absorption, metabolism, distribution, and biotransformation before reaching the ischemic brain. Evidence of systemic exposure or brain accumulation therefore cannot by itself establish a myeloid cell-mediated mechanism. A convincing mechanistic framework requires demonstration that pharmacologically active constituents reach defined myeloid populations, engage the proposed molecular targets within those cells, and induce functional responses that contribute causally to therapeutic benefit.

For multi-component TCM formulas, the interpretation of target engagement is further complicated by variability in chemical composition and systemic exposure. Differences in botanical sources, extraction procedures, constituent concentrations, and manufacturing batches may affect pharmacological reproducibility, making standardized preparation and quantitative chemical profiling essential. Several preclinical studies have begun to characterize formula composition and exposure. High-performance liquid chromatography identified representative constituents of Huoluo Xiaoling Pellet, including salvianolic acid B, ferulic acid, chlorogenic acid, and tanshinone IIA [55]. In studies of Buyang Huanwu Decoction, chemical fingerprinting was combined with the detection of ferulic acid and amygdalin in rat brain tissue following administration [60]. Similarly, analysis of Yiqi Huoxue Formula identified multiple chemical constituents and a subset detectable in rat serum [63]. These findings provide preliminary information on formula composition and systemic or cerebral exposure, but they do not establish the pharmacologically active constituents responsible for myeloid regulation. Moreover, detection in serum or whole-brain tissue does not define the concentrations achieved within microglia or monocyte-derived macrophages. Future investigations should therefore integrate batch-level quality control with quantitative pharmacokinetic analyses of absorption, metabolism, circulating exposure, brain distribution, and clearance. Such analyses should distinguish parent compounds from active metabolites and determine whether achievable concentrations are sufficient for the proposed molecular effects. Importantly, central nervous system penetration is not necessarily required for indirectly mediated effects involving peripheral immune or metabolic pathways. Distinguishing direct central target engagement from peripheral mechanisms will be essential for establishing reproducible and biologically plausible explanations of formula-associated neuroprotection.

Emerging delivery studies illustrate both the potential and the limitations of current evidence. Exosome-like nanoparticles derived from *Panax notoginseng* can reach the ischemic brain and are associated with preservation of blood–brain barrier integrity and a shift toward repair-associated microglial phenotypes, with PI3K/Akt signaling implicated in these effects [74]. These findings support the capacity of medicinal plant-derived nanoparticles to access injured brain tissue and influence central immune responses, but they do not establish selective delivery to microglia. This distinction is reinforced by studies of *Panax notoginseng*-derived extracellular vesicle-like nanoparticles, which can deliver plant microRNAs to the ischemic brain while preferentially targeting neurons rather than microglia in the examined experimental system [75]. Brain accessibility should therefore be distinguished from target-cell accessibility when evaluating the cellular mechanism of TCM-associated delivery systems.

More selective delivery strategies provide a stronger basis for linking therapeutic exposure to defined myeloid populations. Following stroke, p21^+^CD86^+^ microglia accumulate within the injured brain, and targeted delivery of quercetin using a CD86-recognizing peptide preferentially reduces this pathological microglial population. This intervention is accompanied by attenuation of neuroinflammation, preservation of blood–brain barrier integrity, and improved functional recovery [76]. Compared with nonspecific brain delivery, this approach more directly connects therapeutic exposure with a defined microglial population. However, selective cellular targeting alone does not establish molecular target engagement or prove that the resulting microglial change is required for therapeutic benefit. A complete mechanistic framework would therefore need to verify drug accumulation within the relevant cells, engagement of the proposed intracellular target, and a cell-specific functional response that contributes causally to the observed outcome.

A further translational gap separates experimental evidence of myeloid regulation from clinically demonstrated therapeutic benefit. In a multicenter randomized placebo-controlled trial involving 2200 patients, adjunctive Naoxintong Capsule treatment was associated with a reduced risk of recurrent ischemic stroke [77]. A randomized double-blind placebo-controlled trial involving 118 patients with subacute to chronic ischemic stroke further reported improvements in vascular outcomes and selected cognitive and emotional measures after 24 weeks of Jianpi Fuzheng Formula treatment [78]. These trials demonstrate that TCM interventions can be evaluated using standardized clinical endpoints, but they provide no direct evidence that modulation of microglia or monocyte-derived macrophages mediates the observed clinical benefit.

Bridging the gap between experimental myeloid regulation and clinical translation requires an integrated evidence chain linking therapeutic exposure, target-cell accessibility, molecular target engagement, functional myeloid remodeling, and clinically measurable outcomes. Preclinical studies should identify pharmacologically active constituents that reach the circulation and ischemic brain, determine whether they access resident microglia or monocyte-derived macrophages, and verify engagement of proposed molecular targets within these cells. These cell-resolved responses should then be linked to biomarkers that can be measured across experimental and clinical settings. Ultimately, translational validation will require demonstrating that stage-appropriate treatment exposure produces defined myeloid responses that are mechanistically connected to tissue repair and predictive of neurological or vascular outcomes. Such an evidence framework would distinguish causal myeloid mechanisms from parallel changes accompanying broader neuroprotection and provide a more rigorous basis for evaluating the clinical relevance of TCM-mediated myeloid regulation.

## 6. Limitations

This review integrates evidence from experimental and clinical studies to examine traditional Chinese medicine-mediated regulation of post-stroke myeloid responses within a functional remodeling framework. As a narrative review, its literature selection was guided by mechanistic relevance and conceptual coherence rather than a systematic assessment of all published studies. The search primarily relied on PubMed, and relevant evidence from other databases may therefore not have been fully captured. Furthermore, differences in experimental models, intervention protocols, and methods used to characterize myeloid populations limit direct comparisons across studies. Although particular attention was given to the distinction between associative findings and experimentally supported mechanisms, the available evidence remains insufficient to establish a consistent causal relationship between specific myeloid functional states and therapeutic outcomes. Accordingly, the proposed framework should be regarded as an interpretative synthesis of current findings rather than a definitive model of traditional Chinese medicine-mediated neuroprotection.

## 7. Future Perspectives

Future investigations should move beyond descriptive changes in polarization-associated markers to establish the temporal, spatial, and lineage-specific mechanisms underlying traditional Chinese medicine-mediated myeloid regulation after ischemic stroke. Longitudinal single-cell and spatial approaches, combined with lineage tracing and cell-specific functional perturbation, will be essential for determining whether defined microglial or monocyte-derived macrophage responses are required for tissue repair. Particular attention should be given to the relationship between treatment timing, local microenvironmental conditions, and the functional demands of different stages of stroke recovery.

Pharmacological characterization represents another priority for clinical translation. Future studies should identify the bioavailable constituents of traditional Chinese medicine interventions, characterize their metabolic profiles and brain accessibility, and establish whether they reach and engage proposed molecular targets within relevant myeloid populations. These investigations should be integrated with standardized formulation quality control, pharmacokinetic assessment, and functional outcome measurements. Ultimately, carefully designed translational studies will be necessary to connect pharmacological exposure, cell-specific target engagement, myeloid functional remodeling, and clinically meaningful neurological recovery.

## 8. Conclusions

Post-stroke myeloid immunity is characterized by dynamic and multidimensional functional remodeling rather than a linear transition between predefined polarization states. Resident microglia and monocyte-derived macrophages retain distinct developmental origins but exhibit overlapping and context-dependent functional programs shaped by disease stage, spatial distribution, metabolic adaptation, and local inflammatory signals. This functional diversity provides a more informative framework for understanding the contributions of myeloid populations to ischemic injury and tissue repair.

Current evidence suggests that traditional Chinese medicine-associated bioactive compounds and multi-component formulas may influence post-stroke myeloid responses through receptor-mediated sensing, intracellular inflammatory and metabolic signaling, and nuclear transcriptional regulation. However, most mechanistic findings remain derived from cellular and animal studies, and direct evidence of myeloid-specific causal mediation in humans is limited. Establishing the clinical relevance of these interventions will require rigorous validation of pharmacological exposure, target engagement, cellular function, and neurological outcomes. Accordingly, traditional Chinese medicine-mediated myeloid remodeling should be regarded as a promising but incompletely validated therapeutic framework rather than an established mechanism of clinical benefit.

## Figures and Tables

**Table 1:** Representative preclinical studies of TCM-associated bioactive compounds that modulate myeloid responses and related functions after ischemic stroke.

Mechanistic Level	Bioactive Compound	Experimental Model	Cell Types Assessed	Major Regulatory Mechanisms or Pathways	Myeloid Cell States and Functional Outcomes	Evidence Type	Ref.
Receptor-Mediated Sensing	Astragalus polysaccharide	MCAO rat model; OGD-treated BV2 cells	Microglia	ATP/P2X7R	Pro-inflammatory phenotype ↓; repair-associated phenotype ↑; blood–brain barrier integrity ↑	In vivo and in vitro	[31]
	Baicalin	MCAO/R mouse model; OGD/R-treated BV2 cells	Microglia	TREM2	CD86 and iNOS ↓; Arg1 and CD206 ↑; neurological deficits ↓	In vivo and in vitro	[35]
	β-Caryophyllene	Cerebral I/R in wild-type and TLR4-knockout mice; primary microglial model	Microglia	TLR4/MyD88/TRIF	Pro-inflammatory phenotype ↓; repair-associated phenotype ↑; cerebral infarct volume ↓	In vivo and in vitro	[50]
	Acteoside	MCAO rat model; OGD-treated BV2 cells	Microglia	HMGB1/TLR4/NF-κB/NLRP3	CD86 and iNOS ↓; CD206 and Arg1 ↑; blood–brain barrier injury ↓	In vivo and in vitro	[51]
Intracellular Signaling and Metabolic Integration	Tanshinone IIA	MCAO rat model; OGD-treated BV2 cells	Microglia	IκBα/NF-κB p65	CD16/32 and iNOS ↓; Arg1 and CD206 ↑; cerebral infarct volume ↓	In vivo and in vitro	[36]
	Hydroxysafflor yellow A	MCAO/R mouse model; LPS-stimulated BV2 cells	Microglia	SIRT1/HMGB1/NF-κB	CD86, CD16/32, and iNOS ↓; YM1/2, Arg1, and TGF-β ↑; neuroinflammation ↓	In vivo and in vitro	[37]
	Erianin	Cerebral I/R rat model; OGD/R-treated microglial cells	Microglia	PI3K/Akt/NF-κB	Pro-inflammatory phenotype ↓; repair-associated phenotype ↑; cerebral ischemia/reperfusion injury ↓	In vivo and in vitro	[38]
	Aucubin	MCAO rat model	Microglia	AMPK/Nrf2	Nos2 ↓; CD206 and Arg1 ↑; brain tissue injury ↓	In vivo	[39]
	Formononetin	Animal model of cerebral ischemia/reperfusion; OGD/R-treated BV2 cells	Microglia	AMPK/mTOR/HIF-1α	CD16/32 ↓; CD206 ↑; glycolytic activity ↓; cerebral I/R injury ↓	In vivo and in vitro	[40]
	Curcumin	MCAO mouse model; LPS/ATP-stimulated primary microglia	Microglia/macrophages	NF-κB/NLRP3/Caspase-1/GSDMD	Microglial/macrophage pyroptosis ↓; white matter injury ↓; sensorimotor function ↑	In vivo and in vitro	[41]
	Effusol	MCAO/R rat model; OGD/R-treated BV2 cells	Microglia	NLRP3/ASC/Caspase-1/GSDMD	Microglial pyroptosis ↓; brain injury ↓	In vivo and in vitro	[42]
	Salvianolic acid C	MCAO mouse model; LPS/IFN-γ-stimulated BV2 cells; hypoxia-treated bEnd.3 cells	Microglia/brain microvascular endothelial cells	PKM2/glycolysis; TLR4/MyD88	F4/80^+^iNOS^+^ cells ↓; endothelial tube formation ↑; cerebral angiogenesis ↑	In vivo and in vitro	[52]
Nuclear Transcriptional and Epigenetic Regulation	Baicalein	MCAO animal model; ischemic/inflammatory microglial models	Microglia/macrophages	TLR4/NF-κB/STAT1	Pro-inflammatory polarization ↓; repair-associated phenotype ↑; cerebral infarction injury ↓	In vivo and in vitro	[43]
	Ginkgetin	MCAO animal model; OGD-treated primary microglia	Microglia	PPARγ	M1-associated markers ↓; M2-associated markers ↑; neurological functional recovery ↑	In vivo and in vitro	[44]
	Astragaloside IV	MCAO/R rat model	Microglia/macrophages	PPARγ	M1-associated phenotype ↓; M2-associated phenotype ↑; neurovascular repair ↑	In vivo	[9]
	Fraxin	MCAO rat model; OGD-treated BV2 and HT22 cells	Microglia	PPARγ/NF-κB; Nrf2/HO-1	iNOS ↓; Arg1 ↑; cerebral edema ↓	In vivo and in vitro	[45]
	Curcumol	MCAO/R mouse model; microglial model	Microglia	Nrf2/HO-1/NF-κB	Pro-inflammatory polarization ↓; repair-associated phenotype ↑; cerebral infarct volume ↓	In vivo and in vitro	[46]
	Saponins from *Panax notoginseng* (Burkill) F.H.Chen	Photothrombotic stroke mouse model	Microglia	HIF-1α/PKM2/STAT3	Microglial activation and inflammatory responses ↓; ischemic brain injury ↓	In vivo	[48]
	Pterostilbene	MCAO/R mouse model; OGD/R-treated primary microglia	Microglia	HDAC3/Nrf1	iNOS ↓; Arg1 ↑; neuroinflammation ↓; neurological deficits ↓	In vivo and in vitro	[49]
	Tetrahydroxystilbene glucoside	Mouse model of ischemic stroke; LPS/IFN-γ-stimulated RAW264.7 cells	Macrophages	PKM2 conformation/nuclear translocation and metabolic reprogramming	M1-associated markers ↓; M2-associated markers, including CD206 and Arg1, ↑; neurological deficits ↓	In vivo and in vitro	[53]

**Note:** ↑ indicates an increase or promotion, whereas ↓ indicates a decrease or inhibition. Given the dynamic and heterogeneous functional states of microglia and monocyte-derived macrophages after stroke, this table reports polarization-associated markers and functional outcomes as assessed in the original studies rather than reclassifying them into strict M1/M2 categories. Some studies primarily evaluated inflammasome activation, pyroptosis, phagocytosis, blood–brain barrier integrity, or tissue repair without systematically assessing canonical polarization markers. The term TCM-associated indicates that the compounds have documented occurrence or pharmacological relevance in Chinese medicinal materials and does not imply that the compounds used in the cited experiments were directly isolated from those materials or are exclusive to traditional Chinese medicine. Evidence types are classified according to the experimental models employed in the original studies. In vivo refers to animal experiments, whereas in vitro refers to cell-based experimental systems. Studies incorporating both approaches are designated as in vivo and in vitro. These categories describe the experimental source of evidence rather than the strength of mechanistic causality. Although selected studies employed pharmacological inhibition, genetic manipulation, or target engagement assays, such findings do not necessarily establish myeloid cell-specific mediation of therapeutic effects. All studies summarized in this table are preclinical, and their findings should not be interpreted as established therapeutic mechanisms in humans.

**Table 2:** Representative preclinical studies of traditional Chinese medicine formulas and preparations associated with myeloid cell-state regulation and functional outcomes after ischemic stroke.

TCM Formula/Preparation	Composition	Experimental Model	Cell Types Assessed	Major Regulatory Mechanisms or Pathways	Myeloid Cell States and Functional Outcomes	Evidence Type	Ref.
Danhong Injection	Danshen (*Salvia miltiorrhiza* Bunge var. *miltiorrhiza*) and Honghua (*Carthamus tinctorius* L.)	pMCAO mouse model; OGD-treated microglial cells	Microglia	JUNB/NF-κB/ASS1	Pro-inflammatory markers ↓; repair-associated markers ↑; neuroinflammation ↓; neurological deficits ↓	In vivo and in vitro	[54]
Huoluo Xiaoling Pellet	Danshen (*Salvia miltiorrhiza* Bunge), Danggui (*Angelica sinensis* (Oliv.) Diels), Ruxiang (*Boswellia sacra* Flück.), and Moyao (*Commiphora myrrha* (T.Nees) Engl.)	MCAO rat model; OGD/R-treated microglia	Microglia	MCPIP1/PPARγ	CD16 and iNOS ↓; CD206 and Arg1 ↑; inflammatory mediators ↓; neurological deficits ↓	In vivo and in vitro	[55]
Naoqing Formula	Chuanxiong (*Ligusticum striatum* DC.), Sanqi (*Panax notoginseng* (Burkill) F.H.Chen), Shexiang (musk), Bingpian (*Dryobalanops aromatica* C.F.Gaertn.), Bohe oil (*Mentha canadensis* L.), and Shichangpu oil (*Acorus tatarinowii* Schott)	MCAO mouse model; OGD/R- and LPS-induced cellular injury models	Microglia/macrophages	Csf3/JAK2/STAT3/STAT6	CD86 ↓, CD206 ↑; infarct injury ↓	In vivo and in vitro	[56]
Huangqi Guizhi Wuwu Decoction	Huangqi (*Astragalus mongholicus* Bunge), Guizhi (*Neolitsea cassia* (L.) Kosterm.), Baishao (*Paeonia lactiflora* Pall.), Shengjiang (*Zingiber officinale* Roscoe), and Dazao (*Ziziphus jujuba* Mill.)	MCAO rat model	Microglia	SIRT1/NF-κB/NLRP3	CD16/iNOS ↓, CD206/Arg1 ↑; synaptic plasticity ↑	In vivo	[57]
		MCAO rat model; OGD/R-treated primary microglia	Microglia	PI3K/Akt/mTOR	CD86/iNOS ↓, CD206/Arg1 ↑; neurological deficits ↓	In vivo and in vitro	[58]
Sanhua Decoction	Dahuang (*Rheum palmatum* L.), Houpo (*Magnolia officinalis* Rehder & E.H.Wilson), Zhishi (*Citrus assamensis* S.Dutta & S.C.Bhattacharya), and Qianghuo (*Notopterygium tenuifolium* M.L.Sheh & F.T.Pu)	Cerebral I/R rat model; antibiotic-treated pseudo-germ-free rats	Microglia	Gut microbiota-associated metabolic regulation	CD16-associated features ↓; CD206-associated features ↑; inflammatory mediators ↓; BBB integrity ↑	In vivo	[59]
Buyang Huanwu Decoction	Huangqi (*Astragalus mongholicus* Bunge), Chishao (Paeoniae Radix Rubra), Chuanxiong (*Conioselinum anthriscoides* ‘Chuanxiong’), Danggui (*Angelica sinensis* (Oliv.) Diels), Dilong (Pheretima), Taoren (*Prunus persica* (L.) Batsch), and Honghua (*Carthamus tinctorius* L.)	MCAO rat model with delayed t-PA; LPS/ATP-stimulated BV2 cells	Microglia	PGC-1α/NF-κB/NLRP3/GSDMD	Iba1^+^CD16/32^+^ cells ↓; Iba1^+^CD206^+^ cells ↑; pyroptosis ↓; hemorrhagic transformation ↓	In vivo and in vitro	[60]
		tMCAO mouse model	Microglia	TREM2/ERK1/2/IGF1	Phagocytosis-associated markers ↑; microglial IGF1-associated signals ↑; white matter integrity ↑; remyelination-associated outcomes ↑	In vivo	[61]
Taohong Siwu Decoction	Taoren (*Prunus persica* (L.) Batsch), Honghua (*Carthamus tinctorius* L.), Danggui (*Angelica sinensis* (Oliv.) Diels), Chuanxiong (*Conioselinum anthriscoides* ‘Chuanxiong’), Baishao (*Paeonia lactiflora* Pall.), and Shudihuang (*Rehmannia glutinosa* (Gaertn.) Libosch. ex DC.)	tMCAO mouse model	Microglia	Gut microbiota/LPS; TLR4/NF-κB signaling pathway	iNOS^+^ cells ↓; Arg1^+^ cells ↑; neurological function ↑	In vivo	[62]
Yiqi Huoxue Formula	Huangqi (*Astragalus mongholicus* Bunge), Chuanxiong (*Conioselinum anthriscoides* ‘Chuanxiong’), Danggui (*Angelica sinensis* (Oliv.) Diels), and Danshen (*Salvia miltiorrhiza* Bunge)	tMCAO animal model	Microglia	PPARγ	Iba1^+^iNOS^+^ cells ↓; Iba1^+^Arg1^+^ cells ↑; angiogenesis ↑	In vivo	[63]

**Note:** ↑ indicates an increase or promotion, whereas ↓ indicates a decrease or inhibition. pMCAO, permanent middle cerebral artery occlusion; tMCAO, transient middle cerebral artery occlusion; MCAO/R, middle cerebral artery occlusion/reperfusion; OGD/R, oxygen–glucose deprivation/reoxygenation; t-PA, tissue-type plasminogen activator; SCFAs, short-chain fatty acids. Given the dynamic and heterogeneous functional states of microglia and monocyte-derived macrophages after stroke, this table reports polarization-associated markers and functional outcomes as assessed in the original studies rather than reclassifying them into strict M1/M2 categories. In the study of Taohong Siwu Decoction, iNOS and Arg1 were measured separately without double immunostaining with Iba1. The study of Buyang Huanwu Decoction [61] primarily evaluated TREM2-dependent microglial functions, including phagocytosis and IGF1 secretion, rather than canonical polarization markers. For ingredients whose source species were not specified in the original studies, the pharmaceutical or material names reported by the original authors were retained without assigning an inferred species. Evidence types are classified according to the experimental models employed in the original studies. In vivo refers to animal experiments, whereas in vitro refers to cell-based experimental systems. Studies incorporating both approaches are designated as in vivo and in vitro. These categories describe the experimental source of evidence rather than the strength of mechanistic causality. All studies summarized in this table are preclinical, and associations between formula administration, myeloid responses, and tissue repair should not be interpreted as definitive evidence of myeloid cell-specific causal mediation.

## Data Availability

No new data were created or analyzed in this study. Data sharing is not applicable to this article.

## Abbreviations

The following abbreviations are used in this manuscript:

AGE–RAGE	advanced glycation end products–receptor for advanced glycation end products
Akt	protein kinase B
AMPK	AMP-activated protein kinase
Arg1	arginase 1
ASC	apoptosis-associated speck-like protein containing a CARD
ATP	adenosine triphosphate
AXL	AXL receptor tyrosine kinase
BBB	blood–brain barrier
BDNF	brain-derived neurotrophic factor
CCL2	C-C motif chemokine ligand 2
CCR2	C-C motif chemokine receptor 2
CCND1	cyclin D1
CD	cluster of differentiation
CETSA	cellular thermal shift assay
CH25H	cholesterol 25-hydroxylase
CSF3	colony-stimulating factor 3
CX3CL1	C-X3-C motif chemokine ligand 1
DAMPs	damage-associated molecular patterns
DARTS	drug affinity responsive target stability
ERK1/2	extracellular signal-regulated kinases 1 and 2
FOXP3	forkhead box P3
GAP43	growth-associated protein 43
GPNMB	glycoprotein non-metastatic melanoma protein B
GPR35	G protein-coupled receptor 35
GSDMD	gasdermin D
HDAC3	histone deacetylase 3
HIF-1α	hypoxia-inducible factor 1 alpha
HMGB1	high mobility group box 1
HO-1	heme oxygenase 1
I/R	ischemia/reperfusion
Iba1	ionized calcium-binding adaptor molecule 1
ICAM-1	intercellular adhesion molecule 1
IFN-γ	interferon gamma
IGF1	insulin-like growth factor 1
IL	interleukin
iNOS	inducible nitric oxide synthase
IκBα	inhibitor of nuclear factor kappa B alpha
JAK2	Janus kinase 2
JUNB	JunB proto-oncogene, AP-1 transcription factor
LPS	lipopolysaccharide
MafB	MAF bZIP transcription factor B
MAPK	mitogen-activated protein kinase
MCAO	middle cerebral artery occlusion
MCAO/R	middle cerebral artery occlusion/reperfusion
MCPIP1	monocyte chemotactic protein-1-induced protein 1
miRNA	microRNA
MMP-9	matrix metalloproteinase 9
mTOR	mechanistic target of rapamycin
MyD88	myeloid differentiation primary response 88
NF-κB	nuclear factor kappa B
NLRP3	NLR family pyrin domain containing 3
Nrf1	nuclear factor (erythroid-derived 2)-like 1
Nrf2	nuclear factor erythroid 2-related factor 2
OASL	2′-5′-oligoadenylate synthetase-like
OGD	oxygen–glucose deprivation
OGD/R	oxygen–glucose deprivation/reoxygenation
P2X7R	P2X7 purinergic receptor
PGC-1α	peroxisome proliferator-activated receptor gamma coactivator 1 alpha
PI3K	phosphoinositide 3-kinase
PKM2	pyruvate kinase M2
pMCAO	permanent middle cerebral artery occlusion
PPARγ	peroxisome proliferator-activated receptor gamma
PSD95	postsynaptic density protein 95
SCFAs	short-chain fatty acids
SIRT1	sirtuin 1
SPP1	secreted phosphoprotein 1
SPR	surface plasmon resonance
ST2	suppression of tumorigenicity 2
STAT	signal transducer and activator of transcription
TCM	traditional Chinese medicine
TGF-β	transforming growth factor beta
TLR4	Toll-like receptor 4
tMCAO	transient middle cerebral artery occlusion
t-PA	tissue-type plasminogen activator
TREM2	triggering receptor expressed on myeloid cells 2
TRIF	TIR-domain-containing adapter-inducing interferon beta
YY1	Yin Yang 1
ZFP384	zinc finger protein 384
ZO-1	zonula occludens 1
